# Treatment of Axial Spondyloarthritis: What Does the Future Hold?

**DOI:** 10.1007/s11926-020-00924-5

**Published:** 2020-07-20

**Authors:** Denis Poddubnyy, Joachim Sieper

**Affiliations:** 1 Department of Gastroenterology, Infectious Diseases and Rheumatology, Campus Benjamin Franklin, Charité – Universitätsmedizin Berlin, Hindenburgdamm 30, 12203 Berlin, Germany; 2 Department of Epidemiology, German Rheumatism Research Centre, Berlin, Germany

**Keywords:** Axial spondyloarthritis, Ankylosing spondylitis, Treatment

## Abstract

**Purpose of Review:**

To provide a summary of the recent and expected developments related to the treatment of axial spondyloarthritis.

**Recent Findings:**

An increasing number of interleukin-17 blocking agents show efficacy in axial spondyloarthritis including both non-radiographic and radiographic forms. Janus kinase inhibitors showed promising results in phase II studies in radiographic axial spondyloarthritis and have, therefore, a potential to become a therapeutic option in this indication in the future. Inhibition of structural damage progression in axial spondyloarthritis seems to be possible in the case of effective and early anti-inflammatory treatment, although there are still open questions related to particular drug classes.

**Summary:**

Despite major advances in the field and growing therapeutic options, there are still many open questions related to the optimized treatment strategies and to the individual choice of a drug in axial spondyloarthritis.

## Introduction

Axial spondyloarthritis (axSpA) is a term which covers both patients with already obvious structural damage in the sacroiliac joints (SIJ) visible on X-rays, termed radiographic axSpA—r-axSpA (also knowns as ankylosing spondylitis—AS)—and patients who have not yet developed such structural damage in the SIJ, termed non-radiographic (nr)-axSpA [1]. A substantial part of patients with axSpA will move from nr-axSpA to rad axSpA over time, but not all of them [2, 3]. The inflammation normally starts in the SIJ but can later on extend to inflammatory and structural changes in the spine. AxSpA is a disease of young people (starting before the age of 45 years, but often beginning in the third decade of life), and men are slightly more frequently affected than women [4].

Current Assessment of Spondyloarthritis International Society (ASAS)–European League Against Rheumatism (EULAR) [5] and American College of Rheumatology (ACR)–Spondylitis Association of America (SAA)–Spondyloarthritis Research and Treatment Network (SPARTAN) [6] management recommendations cover the whole group of axSpA. First line of medical treatment is still non-steroidal anti-inflammatory drugs (NSAIDs) followed by biological disease-modifying anti-rheumatic drugs (bDMARDs) and—potentially in the future—targeted synthetic (ts) DMARDs such as Janus kinase (JAK) inhibitors (JAKi). Based on these current recommendations, we will address in this review open question about current and future treatment possibilities. We discuss this for axSpA although older studies often included only patients with r-axSpA.

## NSAIDs

### Clinical Efficacy and Safety of NSAIDs

NSAIDs are clinically quite effective for patients’ symptoms with a greater effect in axSpA patients compared with patients with mechanical low back pain, and they are generally regarded as first-line pharmacological treatment for ax pA [5, 6]. However, there are several open questions about how these drugs work, if they have an effect on inflammation beyond symptomatic improvement, and what is their role in a treatment algorithm once a bDMARD has been started. It is believed that their good efficacy is due to their anti-inflammatory capacity, although the exact mechanism why NSAIDs work so well in the treatment of axSpA is still not known. It could also be shown in several studies in AS that NSAIDs can lower C-reactive protein (CRP) [7, 8] and in another axSpA study that NSAIDs can reduce inflammation in the axial skeleton as detected by magnetic resonance imaging (MRI) [9], although the latter study did not include a placebo group.

It can be assumed that, in the beginning of the disease, symptoms (pain, stiffness, fatigue) are mostly caused by inflammation while later on many other non-inflammatory reasons can contribute to the patient’s symptoms. Indeed, a status of partial remission could be achieved in axSpA patients (symptom duration < 3 years) treated with 1000 mg/day of naproxen for 6 months in 35% of patients [10•], while in older AS studies in patients with longstanding disease such a remission rate could only be achieved in 12–15% of patients [8]. However, this study was not controlled by a placebo group. Taken together, it is possible that NSAIDs also have an effect on objective parameters of inflammation; however, this should be proven in a controlled trial, for example 6 weeks of NSAIDs treatment vs. analgesics without anti-inflammatory properties using disease activity scores, CRP, and MRI-inflammation as outcome parameters.

The safety of NSAIDs has been well investigated and has been described in detail for the cardiovascular, the gastrointestinal, and other systems. However, they are conflicting data for axSpA: some studies describe an increase regarding cardiovascular side effects [11] while others describe even a decrease in cardiovascular complications [12] and mortality [13], which might be due the reduction of inflammation and better mobility of patients treated with NSAIDs, which have both a known positive cardiovascular effect. Furthermore, axSpA patients are generally younger than, for example, patients with rheumatoid arthritis or osteoarthritis, and therefore have a smaller cardiovascular or gastrointestinal risks [14]. More studies are wanted here to come to a final conclusion about the potential risk of NSAIDs for the treatment of axSpA.

### Can Treatment with NSAIDs Stop Radiographic Progression?

In some studies, the question was also addressed whether NSAIDs can retard radiographic progression (growth of syndesmophytes in the spine) in AS patients. This could work via suppression of prostaglandins which can have a stimulatory effect on osteoblast. In one study, it was shown that in AS patients treated continuously (every day) over 2 years with NSAIDs (all patients started with a cyclooxygenase (COX)-2 selective inhibitor celecoxib and the majority of patients continued celecoxib treatment all the time), radiographic progression was slower compared with the control group treated with NSAIDs only on demand [15]. However, such a result could not be confirmed in another similar study in which a non-selective COX-inhibitor diclofenac was used as an NSAID and not celecoxib [16]. This brought up the question whether celecoxib and diclofenac (or selective COX-2 and non-selective COX inhibitors in general) have a different effect on radiographic progression or whether NSAIDs just do not have a retarding effect at all. To address this question further we started a prospective randomized controlled study “COmparison of the effect of treatment with NSAIDs added to anti-TNF therapy versus anti-TNF therapy alone on progression of StrUctural damage in the spine over two years in patients with ankyLosing spondylitis” (CONSUL) and first results can be expected in 2021 (ClinicalTrials.gov-ID: NCT02758782). In this study, active r-axSpA patients with risk factor for radiographic spinal progression (presence of syndesmophytes and/or of elevated CRP) started with a tumor necrosis factor inhibitor (TNFi) and those with good treatment response after 3 months remained in the study and were randomized for adding daily celecloxib (at least 200 mg/day) or no NSAIDs at all. Primary outcome is radiographic progression after 2 years, but also the effect of a combined TNFi and NSAIDs treatment on other clinical and inflammatory parameters compared with a TNF-blocker alone group will be analyzed.

## Biologic and Targeted Synthetic DMARDs

### bDMARDs

TNF inhibitors are very effective for the treatment of axSpA patients failing previous NSAID treatment. Five TNF inhibiting agents have been approved for patients with rad axSpA (in the EU, the USA, and many other countries worldwide) and four for nr-axSpA [17] (formal approval studies have not been performed for infliximab which is the reason that this indication is not in the label for this drug) in the EU and many other countries; however, currently only certolizumab pegol is approved for the indication nr-axSpA in the USA. For giving approval for nr-axSpA, the FDA had asked for a placebo-controlled trial over 12 months which until now was only performed for certolizumab pegol among the TNF inhibiting agents [18].

More recently efficacy in axSpA patients was also proven for several IL-17 inhibitors (IL-17i), with a similar level of efficacy for the rheumatic manifestations—only by indirect comparison of the study results, no head to head data yet available. The IL-17i secukinumab has already been approved worldwide for the indication of r-axSpA [19]. Based on positive results of a recent phase 3 trial (ClinicalTrials.gov ID: NCT02696031), secukinumab has just been approved by the US Food and Drug Administration (FDA) to treat patients with active nr-axSpA. This approval follows a similar decision by the European Commission in April this year. For the IL-17 inhibitor ixekizumab, phase 3 trials have been performed for r-axSpA [20] and nr-axSpA [21] that resulted into approval of the drug for r-axSpA (AS) and nr-axSpA in the USA and in the EU.

Interestingly, until now only a small number of targeted therapies have been proven to be effective in axSpA, different from other chronic inflammatory diseases [17]. Although a similar immunological pathway has been postulated for IL-17 and IL23 anti-IL23, therapies—surprisingly—were not effective for axSpA in placebo-controlled trials [22, 23]. The reason for this is not clear; possible explanations have been discussed recently [24•]. Clarifying why some targeted therapies work but others not will be a crucial research topic for the future to understand the pathogenesis of axSpA better [24•].

### tsDMARDs

Three different JAKi—tofacitinib [25•] (pan-JAK inhibitor targeting JAK 1, 2, and 3), filgotinib [26], and upadacitinib [27] (both targeting preferentially JAK 1)—had been shown to be superior to placebo treatment in phase 2 trials, indeed with an efficacy similar to the one seen with TNF- and IL-17-inhibitors. But interestingly, inhibition of JAK 1 and JAK 3 does not affect the two cytokines currently being identified as crucial (based on treatment responses) for the pathogenesis of axSpA–TNF and IL-17. We need more studies (phase 3 programs with the mentioned JAKi are underway now) confirming a good efficacy of JAKi for the treatment of axSpA and also to understand their exact mechanisms of action in axSpA.

Another tsDMARDS, apremilast, directed against phosphodiesterase-4, which showed some efficacy for psoriasis and psoriatic arthritis, was not effective for the treatment of AS (ClinicalTrials.gov ID: NCT00944658).

### Can we Predict Which b- or tsDMARD Fits Best for a Single Patient?

The availability of 3 different groups of drugs being effective for the treatment of axial SpA—TNFi, IL-17i, and probably for the future also JAKi—raises the question whether different patients respond to these different drugs, whether it is possible to identify patients responding to one drug but not to another one, and whether a combination of different targeted therapies would be effective—and acceptable regarding side effects—in selected patients [24, 28]. Until now, no such information is available to guide the clinicians in their decision. Strategy trials are urgently needed to address this issue. In the intervention arm, initial monotherapy (e.g., with a TNFi) can be followed by a monotherapy with a representative of another drug class monotherapy (e.g., IL-17i or JAKi) in the case of insufficient response in the first phase and finally by a combination of two in the case of insufficient response in the second phase. A comparator arm could be either a sequential monotherapy within one drug class or a “usual care” approach with treatment decisions made by a rheumatologist.

### Can Treatment with bDMARDs Inhibit Radiographic Progression?

There is now more or less general agreement that early and long-term suppression of inflammation by TNFi can also retard radiographic progression [29]. Only in AS patients with longstanding disease, a further radiographic progression can be expected in the first 2–4 years. Predictors of radiographic progression are elevated CRP, male sex, presence of syndesmophytes at baseline, smoking [30], and an elevation of the composite score for disease activity (ASDAS—the ankylosing spondylitis disease activity score) [31, 32]. It could also be shown in one study that lowering the ASDAS in the context of TNF-blocker therapy resulted in slower progression [33].

A first analysis of spinal X-rays after 2 years of treatment with the IL-17i secukinumab indicated a rather slow progression rate (compared indirectly with older TNF-blocker trials); however, there was no control group included for this reading of the spinal radiographs [34]. Another reading of the same X-rays—this time mixed with X-rays from a historical control group not treated with a bDMARD—did not show a significant difference between the two groups [35]. Because of this an ongoing head to head trial comparing the effect of secukinumab with adalimumab over 2 years with the primary outcome parameter of radiographic progression is of great interest (ClinicalTrials.gov ID: NCT03259074); interesting secondary outcome parameters of this study will compare the effect of TNFi and IL-17i inhibitors on clinical outcomes.

## Should We Treat axSpA Patients to Target?

Treat-to-target recommendations have been formulated and recently updated for axSpA [36•]. Aim of any medical treatment should be to get the patient free of clinical and serological signs of disease activity and to prevent the development of structural damage (in the context of axSpA, in the axial skeleton). On this background the treatment target for axSpA has been to reach remission (or inactive disease, as measured by the ASDAS, a composite score which contains patient reported outcome parameters plus CRP, of < 1.3). Only if this cannot be achieved, a status of low disease activity is also acceptable (ASDAS < 2.1). However, it still has to be shown that a more strict and regulated approach to patient’s treatment is superior to standard of care patient’s management regarding, for example, reaching remission and on the long run also retardation of radiographic progression. An association between elevated CRP [30] or an elevation of the composite activity index ASDAS could be shown in the past [31]; even an association between a reduction of the ASDAS by treatment with TNF-blockers was shown [33]. But this assumed association has to be confirmed by a controlled “treat-to-target” trial. One controlled study (ClinicalTrials.gov ID: NCT03043846) comparing 1 year of T2T- therapy with standard-of-care treatment has already finished enrollment, and some results can be expected already in 2020. Another study comparing a treat to target strategy that includes IL-17i as a first-line and a TNFi as a second-line therapy with a standard-of-care approach is recruiting now (ClinicalTrials.gov ID: NCT03906136).

## Can We Predict Good Treatment Response in axSpA?

Although we do not know which bDMARD is best for a single patient for his musculoskeletal manifestations (this is different for the extra-musculoskeletal manifestations such as inflammatory bowel disease, psoriasis, uveitis), we have predictors of a good response to TNFi: short symptom duration (or young age), which probably indicates that symptoms are mostly caused by inflammation, and the presence of objective signs of inflammation such as elevated CRP and subchondral bone marrow inflammation on MRI. The presence of HLA-B27 showed in some studies also a predictive value, probably because the diagnosis of axSpA is more reliable in case of a positive HLA-B27 [37–40]. It has still to be shown whether these parameters also predict good responses to IL-17i [41] and JAKi.

## A Correct Diagnosis Is Crucial for a Good Treatment Response

The drugs we use for the treatment of axSpA (NSAIDs and bDMARDs) are targeting inflammation. Thus, it is obvious that this treatment is not effective or less effective if the cause of the back pain is non-inflammatory such as mechanical back pain or non-specific back pain. Furthermore, inflammation is the main cause of symptoms earlier in the course of the disease while later on other non-inflammatory reasons also play a role. Thus, an early and correct diagnosis is crucial for a successful therapy [10, 38]. However, we are currently facing the problem of both under-diagnosis and over-diagnosis of axSpA.

Under-diagnosis is clearly present because there is still a gap between first symptoms and diagnosis of many years [42]. For this it is important that chronic back pain patients with a possible diagnosis of axSpA find their way to the rheumatologist. Several proposals have been published, and several studies have been performed over the last years how to screen in primary care for axial SpA [43]. Focusing on patients with chronic low back pain starting at an age younger than 45 years is mandatory for all screening proposals, combined with one or more parameters characteristic for axSpA such as inflammatory back pain, a positive HLA-B27 or others [44].

But seeing more and more patients with possible axSpA includes also the risk of over-diagnosis for rheumatologist or other physicians dealing with these patients. Indeed, a positive MRI not so different from what we see in axSpA has also been described, for example, in athletes [45] and the problem of over-diagnosis in rheumatic diseases including axSpA has been discussed recently [46]. For making the diagnosis of axSpA patients earlier, it is important to continue the training of the medical community, especially rheumatologists, on the diagnosis and differential diagnosis of axSpA and on the interpretation of MRI findings. For example, as a part of an educational effort, ASAS has developed recently an interactive case library (free access via the ASAS website: www.asas-group.org) with clinical cases of axSpA patients and of patients with differential diagnoses, with a focus on imaging interpretation in the context of clinical findings. Many educational courses have been performed by ASAS in the last 10–15 years worldwide.

It has been an ongoing problem in SpA, and many other diseases, that classification criteria are often used for diagnosis resulting often in a false diagnosis [47]. This problem has been discussed in detail in the past [48], and again in a recent publication that includes case presentations illustrating the differences between the classification and the diagnostic approaches [49]. While classification criteria normally result in a clear yes or no answer, a diagnostic approach is more flexible and has to take into account the relative weight of single diagnostic parameters, the number of parameters being positive and being negative, and the active exclusion of other diagnoses which could also explain a positive finding [49].

## Is Treatment De-escalation Possible in Axial Spondyloarthritis?

It is clinically a very relevant question whether drug treatment can be reduced in patients responding well, especially in those being in remission. The first question to be answered here is whether the NSAID or the biologic should be reduced/stopped. It has been reported that patients being treated with TNFi can reduce their NSAID intake [50] and a combination therapy of infliximab together with naproxen was very effective in patients with early axSpA (partial remission rate of 62%) compared with naproxen alone [10•]; however, in this latter study unfortunately there was no patient group being on infliximab alone. Some more data on the efficacy of TNFi alone compared with a combination of a TNFi plus an NSAID can be expected in the future from the ongoing CONSUL study (ClinicalTrails.gov ID: NCT02758782).

At the moment this question cannot be answered satisfactorily, but an answer will depend on balancing aspects of costs, efficacy, and side effects.

The second question is if the treatment with a bDMARD can be reduced or stopped in patients with axSpA after remission achievement and if yes, how the reduction should be performed taken risks and benefits into account. In one already mentioned study, patients with active early (< 3 years symptom duration) axSpA were treated with infliximab plus naproxen or with naproxen alone for 6 months. Patients reaching ASAS partial remission were followed up for another 6 months and randomized to receive naproxen 1000 mg daily or no drug at all [51]. After these 6 months a similar proportion was still in remission: 47.5% vs 40.0%, respectively, and a high percentage were still in a status of low disease activity (BASDAI < 3) in both groups: 93.8% vs 87.1%, respectively. Thus, a high proportion of patients treated early did well when the drugs were stopped and whether the NSAID was continued or not did not make a difference. However, follow-up was not continued beyond the 6 months and it might be possible that relapses occurred later [52]. In a more recent study, patients with nr-axSpA were treated with adalimumab and those who reached sustained remission after 26 weeks were randomized for no treatment at all (placebo) or continuation of adalimumab therapy for 40 weeks [53]. A total of 30% of the adalimumab-treated patients experienced a flare while this was the case for 53% in the placebo group (*p* < 0.0001) indicating that (1) continuation of the therapy is associated with a higher chance of remission maintenance, (2) there are patients retaining their remission state despite discontinuation of a bDMARD, and the chance for the sustained remission seems to be higher in early disease. There is still an unmet need for identification of patients in which the treatment can be safely discontinued.

The above-mentioned studies did not address the question whether the dose of the biologic can be reduced without losing the status of remission. This can probably be answered in the near future by the results from 2 studies. In the first study, patients with active axSpA were treated with the TNF-blocker certolizumab pegol for 48 weeks and those patients reaching sustained remission were then randomized for treatment with placebo, certolizumab pegol 200 mg s.c. every 2 weeks (a dosing regimen approved for this indication) or 200 mg s.c. every 4 weeks (ClinicalTrails.gov ID: NCT02505542). Study results might become available in summer 2020. In a second similar study, patients were treated with the TNF-blocker golimumab and those reaching remission were randomized for further placebo, golimumab 50 mg s.c. once a month, and golimumab 50 mg s.c. every second month (ClinicalTrails.gov ID: NCT03253796). Results of this study can be expected in 2021.

## Novel Treatment Options?

There are continuous scientific efforts towards identification of new treatment targets in axSpA. Targeting gut microbiota and restoration of immune tolerance (e.g., with IL-2 or with mesenchymal stem cells) as well as targeted depletion of disease related T cells represent potential future developments we should observe in the next years. The role of a dietary intervention including fasting should be addressed as well.

## Conclusion

We are facing today an increasing number of treatment options in axSpA that generate many clinically relevant questions related to the choice of the best possible treatment strategy in an individual patient (Table 1). They include the choice of the first- and second-line drugs, criteria for treatment escalation and de-escalation, and the possibility of combination therapy. Inhibition of structural damage progression represents another clinically relevant cluster of questions. There are ongoing clinical trials aimed at answering some of those questions, but further clinical trials evaluating different treatment strategies not addressed so far are urgently needed.

**Table 1:** Open questions about treatment of axSpA to be addressed in the future

Treatment	Question	Potential solution
NSAIDs	Effect on objective signs of inflammation, specifically on MRI inflammation	Controlled study with NSAIDs vs analgesics without anti-inflammatory properties
	Effect of NSAIDs in general and of COX-2-selective inhibitors specifically on radiographic spinal progression	Controlled clinical trial, at least 2 years duration with conventional X-rays, potentially shorter with a low-dose computed tomography
	Long-term safety of NSAIDs in axSpA	Long-term registry data
b- und tsDMARDs	Comparative efficacy of TNFi, IL17i and JAK	Head-to-head studies
	The optimal first- and second-line treatment, potential for combination, optimized individual treatment strategy, selective predictors of response	Strategy trials, trials with cross-over design
	Short- and long-term advantages of a treat-to-target strategy, optimal time points and criteria for the escalation of treatment	Trials with the treat-to-target approach
	Optimal strategy for the treatment de-escalation, predictors of sustained drug-free remission	Trials with treatment de-escalation strategies
	Effects of b-and tsDMARDs on radiographic spinal progression, alone or in combination with NSAIDs	Controlled trials, at least 2 years duration with conventional X-rays, potentially shorter with a low-dose computed tomography
Potential novel treatment options	Targeting microbiota, restoration of immune tolerance, targeting disease-relevant T cells, dietary intervention	Identification of the appropriate targets and conduction of trials
